# Geographically linked risk factors for enrolment into a fast breathing child pneumonia trial in Lilongwe, Malawi: an Innovative Treatments in Pneumonia (ITIP) secondary analysis

**DOI:** 10.1136/bmjresp-2019-000414

**Published:** 2019-05-22

**Authors:** Evangelyn Nkwopara, Robert Schmicker, Tisungane Mvalo, Susanne May, Amy Sarah Ginsburg

**Affiliations:** 1 International Programs, Save the Children Federation, Fairfield, Connecticut, USA; 2 Biostatistics, University of Washington, Seattle, Washington, USA; 3 University of North Carolina Project, Lilongwe, Central Region, Malawi

**Keywords:** pneumonia

## Abstract

**Background:**

Pneumonia is the leading infectious killer of children less than 5 years of age worldwide. In addition to vaccines that help prevent pneumonia, understanding the environmental and socioeconomic risk factors for child pneumonia is critical to further prevention.

**Methods:**

Data from children with fast breathing pneumonia enrolled in a non-inferiority clinical trial assessing the effectiveness of 3-day placebo versus antibiotic treatment in Lilongwe, Malawi were used to examine environmental and socioeconomic characteristics within the study population. Location of residence was collected for enrolled children, and spatial enrolment rates were compared across Lilongwe using a spatial scan statistic.

**Results:**

Data from 1101 children were analysed. Three urban subdistricts (locally known as ‘Areas’) (Areas 24, 36 and 38) out of 51 were identified with higher than expected enrolment. These three areas were associated with higher rates of poverty (37.8% vs 23.9%) as well as informal settlements and poorer sanitation (42.4% vs 7.4%) than other areas. Parents of enrolled children from these areas also had lower rates of secondary education compared with parents of children enrolled from other areas (55% vs 67% (p<0.01) among fathers; 47% vs 54% (p<0.01) among mothers).

**Conclusion:**

In Lilongwe, areas with higher rates of poverty, informal settlements and poor sanitation contributed higher than expected enrolment of children to our fast breathing child pneumonia clinical trial when compared with other areas. Additional research is needed to evaluate the impact of environmental and socioeconomic risk factors, along with vaccination status, on the incidence of fast breathing pneumonia in children living in this region.

**Key messages**

- Does residence mapping of children enrolled in a fast breathing pneumonia clinical trial in Lilongwe, Malawi help explain environmental and socioeconomic risk factors for the incidence of childhood fast breathing pneumonia in this region?
- Urban subdistricts in Lilongwe characterised by poverty, informal settlements and poor sanitation contributed to higher than expected enrolment in our childhood fast breathing pneumonia clinical trial.
- Using residence mapping in this clinical trial population in Lilongwe provides insights into environmental and socioeconomic risk factors for childhood pneumonia.

## Introduction

Pneumonia kills approximately 920 000 children under 5 years of age worldwide, over half in Africa.^1^ Significant progress has been made in preventing pneumonia with the introduction of vaccines, specifically *Haemophilus influenzae* type b and *Streptococcus pneumoniae* conjugate vaccines.^2^ However, immunisation, while the most effective intervention for preventing pneumonia, does not prevent all episodes of pneumonia.^3^ Understanding the environmental and socioeconomic contributors that increase the risk of pneumonia infection and disease may be instrumental in bolstering implemented preventative measures such as immunisation. Child pneumonia is a common disease of poverty and poor access to care, with higher rates of child pneumonia-related mortality occurring in low-income and middle-income countries.^4 5^ Previous research has shown an association between pneumonia, poverty and other socioeconomic risk factors in Asia.^6 7^ In this secondary analysis of the Innovative Treatments in Pneumonia (ITIP) 1 trial, a randomised controlled, non-inferiority trial to assess the effectiveness of no antibiotic treatment for fast breathing child pneumonia, we sought to conduct a geospatial analysis of the study’s enrolment data by examining the home residence for enrolled children within the trial’s catchment area of Lilongwe, Malawi, and the variability in socioeconomic status. We endeavoured to explore the association between environmental and socioeconomic factors with child pneumonia in this region. To our knowledge, this type of analysis had not been done previously in Lilongwe, Malawi.

## Methods

### Study design

The ITIP1 fast breathing study was a prospective, double-blind, randomised controlled, two-arm, non-inferiority trial to determine whether or not treatment with placebo in HIV-uninfected children 2–59 months of age with non-severe fast breathing pneumonia (table 1) in Lilongwe, Malawi was less effective than 3 days of treatment with amoxicillin. Children who met the eligibility criteria were enrolled from June 2016 through May 2017 at Kamuzu Central Hospital (KCH) and Bwaila District Hospital (BDH). Primary trial results have been presented elsewhere.^8^

**Table 1:** Study definitions

Area	A subdistrict within Lilongwe, Malawi.
Non-severe fast breathing pneumonia	Cough <14 days or difficulty breathing and fast breathing for age.
Fast breathing for age	Respiratory rate >50 breaths per minute (for children 2 to <12 months of age) or >40 breaths per minute (for children >12 months of age).
Treatment failure criteria	
Any time on or before day 4	Severe respiratory distress (grunting, nasal flaring, head nodding and/or chest indrawing). Hypoxaemia (arterial oxyhaemoglobin saturation <90% in room air, as assessed non-invasively by a pulse oximeter). General danger sign (lethargy or unconsciousness, convulsions, vomiting everything, inability to drink or breast feed, stridor while calm). Missing ≥2 study drug doses due to vomiting. Change in antibiotics prescribed by a study clinician. Hospitalisation due to pneumonia (if not initially admitted). Prolonged hospitalisation or readmission due to pneumonia (if initially admitted). Death.
On day 4 only	Axillary temperature ≥38°C in the absence of a diagnosed coinfection with fever symptoms (eg, malaria).
Clinical relapse criteria	
Any time after day 4	Recurrence of signs of pneumonia. Signs of severe disease.

For the purposes of this secondary analysis, we examined the home residence location of all enrolled children, specifically the spatial enrolment and enrolment rate per 10 000 children.

### Study population

**Figure 1:**
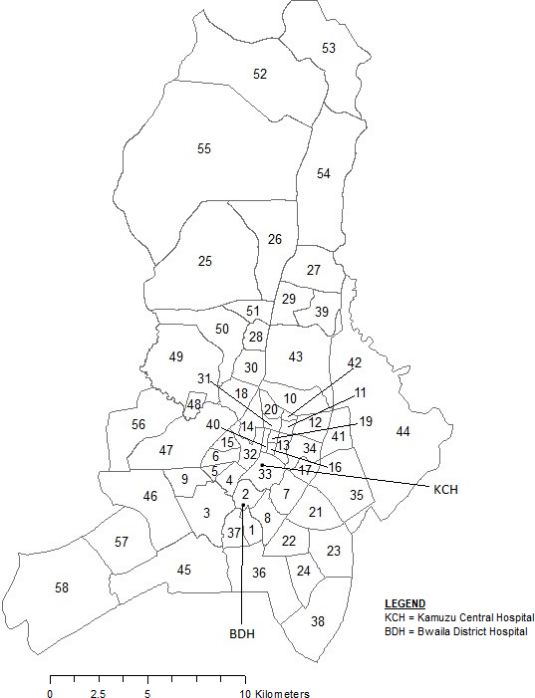
Map of areas in Lilongwe, Malawi.

Malawi is divided into three regions: northern, central and southern. There are a total of 28 districts within these three regions, and these districts are further divided into subdistricts referred to locally as ‘Areas’ (table 1). Enrolment occurred at KCH (located in Area 33) and BDH (located in Area 2), the two largest public healthcare facilities in the capital city of Lilongwe, serving approximately five million people. KCH is approximately 4.0 km (2.5 miles) from BDH. KCH is a tertiary referral centre for the central region of Malawi and has 20 000–25 000 admissions of children per year with about 18 000 admissions for children under 5 years of age.^9^ BDH provides outpatient services for children, and paediatric patients presenting to BDH requiring hospitalisation are transferred to KCH. There are also 4 other hospitals and 30 private and public health centres serving Lilongwe.^10^ The catchment area for this fast breathing pneumonia trial was Lilongwe city, not including surrounding rural Lilongwe. The city of Lilongwe consists of 58 distinct areas (total size of 375.1 km^2^, areas ranging from 0.2 to 60.1 km^2^) and represents over 80% of Malawi’s central region (figure 1). As population estimates for each of the 58 areas were not available for 2016–2017, data were extrapolated from the 2008 Population and Housing Census results for children 0–59 months of age.^11^ Specifically, area population proportions for the city of Lilongwe and individual age group proportions from the central region were applied. While not precisely matching the age criteria for the trial (2–59 months of age), the number of children up to 2 months of age was relatively small (<3% of total). We then extrapolated the estimated 2008 area proportions to 2015 using the 2015 Lilongwe population of 1 037 294.^12^

### Statistical analysis

Absolute enrolment in this trial and estimated population per area are provided in table 2. To determine which areas had significantly higher rates of trial enrolment than expected, we used a spatial scan statistic through SaTScan, whose full methods are described in length elsewhere.^13^ In brief, we first determined the geographical centre of each area, and attributed both the overall population of children up to 59 months of age in that area and the number enrolled in the trial from that area. To create all potential clusters of areas, circles of increasing size were drawn at every centre point, gradually encompassing centre points representing other areas. For each cluster, the rate of enrolment per population was calculated and then compared with the overall rate for the population. The cluster of areas with a rate that was least likely to be due to chance (via likelihood ratio test) was identified as being the most significant. This method was repeated four times, as we varied the maximum population allowed in each cluster. Areas identified in all four iterations were considered the ‘core cluster’.

**Table 2:** Fast breathing pneumonia enrolment and estimated paediatric population by area* in Lilongwe

Area	Total population	Aged 0–5 years population	Trial enrolment (rate per 10 000)	Area	Total population	Aged 0–5 years population	Trial enrolment (rate per 10 000)
57	91 979	20 722	241 (116.3)	46	4918	920	11 (119.6)
25	101 003	18 005	2 (1.1)	3	6876	873	11 (126.0)
36†	72 759	16 128	312 (193.4)	10	4905	774	0 (0.0)
23	70 142	13 233	31 (23.4)	27	3434	756	1 (13.2)
56	56 355	13 088	86 (65.7)	2	4677	687	1 (14.6)
21	68 847	12 693	2 (1.6)	30	3582	600	0 (0.0)
50	41 343	10 470	2 (1.9)	43	3548	564	1 (17.7)
7	61 467	10 020	2 (2.0)	33	3839	501	0 (0.0)
44	39 851	9167	0 (0.0)	12	3554	497	3 (60.4)
22	45 306	8015	34 (42.4)	9	2870	441	8 (181.4)
24†	34 925	6880	210 (305.2)	15	2416	224	0 (0.0)
49	39 233	6755	17 (25.2)	6	3842	208	2 (96.2)
58	36 761	6596	50 (75.8)	14	1779	206	0 (0.0)
8	40 371	6317	7 (11.1)	11	1438	177	0 (0.0)
55	23 847	5102	0 (0.0)	37	854	121	0 (0.0)
51	21 084	4869	1 (2.1)	29	829	118	0 (0.0)
53	28 194	4847	0 (0.0)	5	1161	108	0 (0.0)
38†	14 969	3517	40 (113.7)	45	261	42	1 (238.1)
1	21 046	3086	11 (35.6)	32	532	22	0 (0.0)
18	21 443	2170	1 (4.6)	4	145	20	0 (0.0)
35	11 233	1924	0 (0.0)	42	20	5	2 (4000)
26	7455	1718	0 (0.0)	28	74	4	0 (0.0)
39	7213	1535	0 (0.0)	34	71	4	0 (0.0)
47	12 518	1450	3 (20.7)	20	22	2	0 (0.0)
54	7221	1352	9 (66.6)	40	12	2	0 (0.0)
52	5026	1003	0 (0.0)				

*Areas 13, 16, 17, 19, 31, 41 and 48 had estimated no children 0–5 years old.

†Represents core clusters.

For the purposes of this analysis, and not the trial itself, we did not include enrolled children from the northernmost Areas 52–55 (n=9) since their distance from KCH and BDH (>10 km) could be a deterrent for seeking care, and from Areas 25–27 (n=3) which are served by a healthcare centre that was not involved in the trial. In addition, we did not consider 13 enrolled children who lived outside of the city of Lilongwe and therefore outside of the trial catchment area.

We compared the baseline characteristics, sociodemographic data and environmental exposure data collected for enrolled children who live in the core cluster with the enrolled children who live in the non-core cluster. In addition, we compared population-level data obtained from both the Study on Urban Development Master Plan for Lilongwe in the Republic of Malawi (SUDMP) and the Malawi Poverty and Vulnerability Assessment by core cluster status.^14 15^ Specifically, we compared the percentage of land designated as unplanned or informal settlements (defined by the SUDMP as being illegal housing outside lawfully planned residential areas) and the percentage of residents living below the poverty line for each area.^14 15^ Finally, we compared primary trial outcome measures including rates of treatment failure prior to or on day 4 and clinical relapse occurring after day 4 through day 14 (table 1), by core cluster status. Effect modification by treatment group was then explored with a χ^2^ test. Due to the exploratory nature of this research, p values were not adjusted for multiple comparisons.

### Patient and public involvement

Study participants were not involved in the development of the research question, study design or outcome measures.

## Results

**Figure 2:**
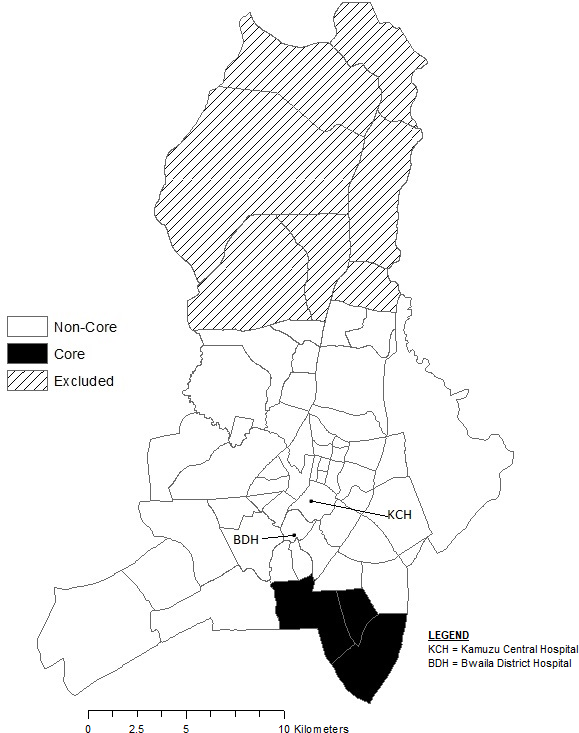
Map of trial core clusters in Lilongwe, Malawi.

There were 1126 children enrolled in the trial, of whom 1101 were included in this analysis. Areas 36, 57 and 24 enrolled 28%, 22% and 19%, respectively. Using a spatial scan statistic, Areas 24, 36 and 38 were considered the core cluster (n=562, 51%) as they were identified as areas with higher than expected enrolment across all four iterations. Within this core cluster, the enrolment rate was 212 per 10 000 children compared with 63 per 10 000 children across all areas. These three densely populated areas are south-east of KCH and BDH (shaded black in figure 2).

When comparing children from the core cluster with those from the non-core cluster, there were no significant differences in average age, gender and proportion with moderate malnutrition (11.5–13.5 cm mid-upper arm circumference, or −2 to −3 height for weight z-score) status, nor were there any differences in average respiratory rate, oxygen saturation and temperature at enrolment (table 3). The core cluster had significantly higher (p<0.02) rates of children who received the age-appropriate number of doses (eg, two doses of pneumococcal conjugate vaccine and pentavalent vaccine required for children 2–3 months; three doses of pneumococcal conjugate vaccine and pentavalent vaccine required for children 4–59 months) of the pneumococcal conjugate (n=350, 62.3%) and pentavalent (n=353, 62.8%) vaccines compared with those in the non-core cluster (pneumococcal conjugate vaccine, n=300, 55.7%; pentavalent vaccine, n=297, 55.1%) (table 4). Although there was a large number of children with all vaccine doses unknown in both the core cluster (33.6%) and the non-core cluster (39.7%), this was almost exclusively among those 4–59 months of age. Only one child 2–3 months of age, in the non-core cluster group, had all doses unknown.

**Table 3:** Enrolled child characteristics at trial enrolment by core cluster

	Core cluster	Non-core cluster	Total
	n=562	n=539	N=1101
Age, n (%)			
2–11 months	195 (34.7)	188 (34.9)	383 (34.8)
12–23 months	155 (27.6)	133 (24.7)	288 (26.2)
24–59 months	212 (37.7)	218 (40.4)	430 (39.1)
Gender, n (%)			
Male	261 (46.4)	254 (47.1)	515 (46.8)
Female	301 (53.6)	285 (52.9)	586 (53.2)
Height/Weight z-score, n (%)			
<−3	0 (0.0)	1 (0.2)	1 (0.1)
−2 to −3	6 (1.1)	7 (1.3)	13 (1.2)
>−2	556 (98.9)	530 (98.5)	1086 (98.7)
Mid-upper arm circumference, n (%)			
<11.5 cm	0 (0.0)	0 (0.0)	0 (0.0)
11.5–13.5 cm	40 (7.1)	36 (6.7)	76 (6.9)
>13.5 cm	522 (92.9)	502 (93.3)	1024 (93.1)
Respiratory rate (breaths/min)*			
Age 2–11 months, n (%)			
<50	1 (0.2)	0 (0.0)	1 (0.1)
50–59	139 (24.7)	132 (24.5)	271 (24.6)
≥60	55 (9.8)	56 (10.4)	111 (10.1)
Age 12–59 months, n (%)			
<40	0 (0.0)	0 (0.0)	0 (0.0)
40–49	232 (41.3)	221 (41.0)	453 (41.1)
≥50	135 (24.0)	130 (24.1)	265 (24.1)
Oxygen saturation, n (%)			
<90%	0 (0.0)	0 (0.0)	0 (0.0)
90%–92%	0 (0.0)	0 (0.0)	0 (0.0)
≥93%	562 (100.0)	539 (100.0)	1101 (100.0)
Axillary temperature, n (%)			
<38°C	397 (70.6)	380 (70.5)	777 (70.6)
≥38°C	165 (29.4)	159 (29.5)	324 (29.4)

*One child, without fast breathing for age, was inappropriately enrolled to the study.

**Table 4:** Vaccination by core cluster

	Core cluster		Non-core cluster		Total	
	2–3 months*	4–59 months*	2–3 months*	4–59 months*	2–3 months*	4–59 months*
	n=35	n=527	n=37	n=502	n=72	n=1029
Pneumococcal conjugate vaccine, n (%)						
Received 3 doses†	4 (11.4)	323 (61.3)	7 (18.9)	274 (54.6)	11 (15.3)	597 (58.0)
Received 2 doses†	2 (5.7)	10 (1.9)	0 (0.0)	12 (2.4)	2 (2.8)	22 (2.1)
Received 1 dose†	0 (0.0)	0 (0.0)	0 (0.0)	0 (0.0)	0 (0.0)	0 (0.0)
Received 0 dose†	0 (0.0)	3 (0.6)	0 (0.0)	0 (0.0)	0 (0.0)	3 (0.3)
All doses unknown	0 (0.0)	189 (35.9)	1 (2.7)	213 (42.4)	1 (1.4)	402 (39.1)
Some doses unknown, n (%)						
Received 2 doses	21 (60.0)	2 (0.4)	19 (51.4)	1 (0.2)	40 (55.6)	3 (0.3)
Received 1 dose	8 (22.9)	0 (0.0)	10 (27.0)	2 (0.4)	18 (25.0)	2 (0.2)
Received 0 dose	0 (0.0)	0 (0.0)	0 (0.0)	0 (0.0)	0 (0.0)	0 (0.0)
Pentavalent vaccine, n (%)						
Received 3 doses†	4 (11.4)	326 (61.9)	7 (18.9)	271 (54.0)	11 (15.3)	597 (58.0)
Received 2 doses†	2 (5.7)	7 (1.3)	0 (0.0)	15 (3.0)	2 (2.8)	22 (2.1)
Received 1 dose†	0 (0.0)	0 (0.0)	0 (0.0)	0 (0.0)	0 (0.0)	0 (0.0)
Received 0 dose†	0 (0.0)	3 (0.6)	0 (0.0)	0 (0.0)	0 (0.0)	3 (0.3)
All doses unknown	0 (0.0)	189 (35.9)	1 (2.7)	212 (42.2)	1 (1.4)	401 (39.0)
Some doses unknown, n (%)						
Received 2 doses	21 (60.0)	2 (0.4)	19 (51.4)	1 (0.2)	40 (55.6)	3 (0.3)
Received 1 dose	8 (22.9)	0 (0.0)	10 (27.0)	3 (0.6)	18 (25.0)	3 (0.3)
Received 0 dose	0 (0.0)	0 (0.0)	0 (0.0)	0 (0.0)	0 (0.0)	0 (0.0)

*Two doses of pneumococcal conjugate vaccine and pentavalent vaccine required for children 2–3 months. Three doses of pneumococcal conjugate vaccine and pentavalent vaccine required for children 4–59 months.

†All dosage information is confirmed.

Both fathers and mothers had significantly lower rates of secondary education in the core cluster than in the non-core cluster (father, core 55% vs non-core 67% (p<0.01); mother, core 47% vs non-core 54% (p<0.01); table 5). There were no significant differences with respect to average monthly income, child care outside of the home, source of drinking water or household smoke exposure across core cluster status.

**Table 5:** Child and parent characteristics and environmental exposure by core cluster

	Core cluster	Non-core cluster	Total	P value
	n=562	n=539	N=1101	
Mean birth weight (SD)	3.1 (0.6)	3.2 (0.6)	3.1 (0.6)	0.06
Mother HIV-positive, n (%)	15 (2.7)	14 (2.6)	29 (2.6)	0.99
Child HIV-positive, n (%)	3 (0.5)	0 (0.0)	3 (0.3)	–
Mean mother age (SD)	25.6 (5.1)	25.6 (5.4)	25.6 (5.2)	0.99
Mother’s highest education level, n (%)				<0.01
None	7 (1.2)	14 (2.6)	21 (1.9)	
Primary	278 (49.5)	215 (39.9)	493 (44.8)	
Secondary	263 (46.8)	289 (53.6)	552 (50.1)	
Tertiary	14 (2.5)	19 (3.5)	33 (3.0)	
Unknown	0 (0.0)	2 (0.4)	2 (0.2)	
Father’s highest education level, n (%)				<0.01
None	4 (0.7)	5 (0.9)	9 (0.8)	
Primary	156 (27.8)	101 (18.7)	257 (23.3)	
Secondary	307 (54.6)	360 (66.8)	667 (60.6)	
Tertiary	51 (9.1)	49 (9.1)	100 (9.1)	
Unknown	44 (7.8)	24 (4.5)	68 (6.2)	
Parents’ monthly income (MK), median (IQR)	40 0000 (22 000–70 000)	42 5000 (25 000–64 000)	40 0000 (25 000–70 000)	0.37
Mean number of children 0–10 years living in the same household (SD)	1.8 (0.9)	1.8 (1.0)	1.8 (0.9)	0.94
Child attends out-of-home care, n (%)	126 (22.4)	151 (28.0)	277 (25.2)	0.04
Any children in the house attend out-of-home care, n (%)	299 (53.2)	280 (51.9)	579 (52.6)	0.75
Smoker in the same household, n (%)	48 (8.5)	59 (10.9)	107 (9.7)	0.21
Main source of drinking water, n (%)				0.50
Outside piped water	473 (84.2)	463 (85.9)	936 (85.0)	
Inside piped water	17 (3.0)	21 (3.9)	38 (3.5)	
Tube/deep tube well	21 (3.7)	16 (3.0)	37 (3.4)	
Surface well/other well	16 (2.8)	17 (3.2)	33 (3.0)	
Other	34 (6.0)	22 (4.1)	56 (5.1)	
Caregiver used soap in the last 24 hours, n (%)	473 (84.2)	467 (86.6)	940 (85.4)	0.28
Child sleeps under a mosquito net, n (%)				0.45
Always	482 (85.8)	466 (86.5)	948 (86.1)	
Sometimes	30 (5.3)	36 (6.7)	66 (6.0)	
Never	33 (5.9)	22 (4.1)	55 (5.0)	
Cooking/heating produces smoke in the household, n (%)	541 (96.3)	507 (94.1)	1048 (95.2)	0.12
Tuberculosis exposure, n (%)	3 (0.5)	4 (0.7)	7 (0.6)	–

MK, Malawi kwacha.

At the population level, the core cluster (42.4%) had a significantly higher percentage of land used for informal settlements in 2008 compared with the non-core cluster (7.4%; p<0.001). Population within the core cluster increased by 9.4% compared with 3.7% in the non-core cluster from 1998 to 2008. Areas 36 and 38 are in the top quartile of the percentage of Lilongwe residents living below the poverty line (44.3%).

Treatment failure prior to day 4 in the core cluster was 6.0% compared with 5.0% among children living in the non-cluster (p=0.54; table 6). When examined by treatment group, treatment failure among those in the amoxicillin group from the core cluster areas was 3.6% compared with 4.5% among children in the amoxicillin group from the non-core cluster. For children in the placebo group, those from the core cluster had a treatment failure rate of 8.5% compared with 5.4% from the non-core cluster (interaction p=0.15). The clinical relapse rate among those who did not have a treatment failure on or by day 4 in the core cluster was 4.9% compared with 8.3% in those from the non-core cluster (p=0.06). Combined, the rate of treatment failure or clinical relapse on or prior to day 14 was 11.2% in the core cluster compared with 13.5% in the non-core cluster (p=0.33).

**Table 6:** Trial outcome by treatment group and core cluster

	Core cluster	Non-core cluster	Total
	n=548	n=523	n=1071
Treatment failure on or before day 4	33 (6.0%)	26 (5.0%)	59 (5.5%)
Amoxicillin group	10 (3.6%)	12 (4.5%)	22 (4.1%)
Placebo group	23 (8.5%)	14 (5.4%)	37 (7.0%)
Clinical relapse after day 4	23 (4.9%)	36 (8.3%)	59 (6.6%)
Amoxicillin group	11 (4.5%)	22 (10.0%)	33 (7.1%)
Placebo group	12 (5.4%)	14 (6.6%)	26 (6.0%)
Treatment failure or clinical relapse on or prior to day 14	56 (11.2%)	62 (13.5%)	118 (12.3%)
Amoxicillin group	21 (8.3%)	34 (14.6%)	55 (11.3%)
Placebo group	35 (14.2%)	28 (12.4%)	63 (13.3%)

## Discussion

An individual’s health and where they live are intrinsically linked. Using geospatial technology to look at population health makes this link more tangible and may reveal some of the drivers for specific health indicators or outcomes as well as how best to address them. This approach has been applied in various regions to gain insight into factors impacting risk for pneumonia and malaria.^16 17^ In Brazil the relationship between paediatric admissions for severe pneumonia with pleural effusion and the Municipal Human Development Index (M-HDI) of children’s places of residence was examined to assess the impact of M-HDI on the incidence of the disease.^18^ In a similar manner, we have attempted to use available demographic, environmental and socioeconomic data for Lilongwe, Malawi, combined with spatial statistics to provide some geographical context to our fast breathing pneumonia trial and gain additional perspective on the possible impact of environment and socioeconomic status on a child’s pneumonia risk. When examining the home residence location of children enrolled in this fast breathing pneumonia trial, Areas 24, 36 and 38 had higher than expected enrolment given the overall population of children living in these three areas. Of interest, Area 57 enrolled the second highest number of children; however, we decided not to include Area 57 as part of the core cluster because it is geographically isolated from the other areas within the core cluster.

Per the SUDMP in the Republic of Malawi, Areas 24, 36 and 38 have a high percentage of unplanned informal settlements that formed because residents are unable to afford housing in planned areas. These informal settlements are characterised by inadequate housing (size, crowding and material construction) and lack of public services.^14^ These settlements are not served by government agencies, and the residents in these areas are exposed to environmental pollution and poor sanitation maintenance. Additionally, Areas 36 and 38 are home to a large portion of Lilongwe residents living below the poverty line.^15^ Areas 24, 36 and 38 also have significantly lower rates of secondary education for mothers and fathers.

The above suggests that in Lilongwe, for children enrolled in the trial from the identified core cluster, these specific environmental and socioeconomic indicators are associated with higher rates of fast breathing pneumonia. This is consistent with studies conducted in South and South-East Asia that found similar relationships between pneumonia and low socioeconomic status, low maternal education rates, crowding and poor sanitation.^19–21^ In addition to an association with higher study enrolment, children from the core cluster fared worse initially in the trial when compared with those in the non-core cluster, with a slightly higher, non-significant rate of treatment failure (core, 6.0% treatment failure within 4 days; non-core, 5.0% treatment failure within 4 days), and then later had a slightly lower, non-significant rate of treatment failure or clinical relapse on or prior to day 14 (core, 11.2%; non-core, 13.5%; p=NS).

A limitation to this secondary analysis is that it can only provide a limited understanding of the geographical distribution of fast breathing pneumonia among children 2–59 months of age within Lilongwe. While the trial enrolled children presenting to KCH or BDH, the two largest public healthcare facilities in Lilongwe city, this does not account for all children with fast breathing pneumonia presenting to all healthcare facilities within the trial catchment area during this time period. It is likely that a portion of children within the trial catchment area presented and received care from other healthcare facilities not involved in the trial. For example, Kawale Health Center in Area 7 was not involved in the trial and could have received and treated children with fast breathing pneumonia from Areas 7 and 21. Areas 21 and 7 have the sixth and eighth highest population of children up to 5 years of age, respectively, but each enrolled only two children in the trial. The effect of potential missed enrolment is unknown. Another limitation is that enrolment primarily occurred between the hours of 08:00 and 12:00, which means that if children were coming from further away within the catchment area or simply at different times, they could have been missed if they arrived to KCH or BDH later in the day. A further limitation is that more up-to-date and detailed population-level data for Lilongwe were not available at the time of this analysis. In this analysis, population estimates in each area were extrapolated from 2008 using a constant factor consistent with the overall increase in Lilongwe population from 2008 to 2015.

As noted previously, this was a secondary analysis and the trial was not designed to examine possible risk factors in Lilongwe for fast breathing pneumonia. However, this type of analysis highlights the need for further research specifically exploring the impact of environmental exposures and socioeconomic indicators associated with rates of fast breathing pneumonia in malaria-endemic settings in Africa, such as Malawi. The results from this secondary analysis may be useful in improving national-level polices to achieve a more targeted and integrated approach to pneumonia prevention. For example, it appears that immunisation efforts in our identified core cluster may not be sufficient in preventing the spread of disease. Perhaps, the large number of children overall with an unknown immunisation status, coupled with poor sanitation and pollution, indicates that some additional interventions, including possible increased immunisation coverage, are necessary within the core cluster to further bolster current pneumonia prevention efforts.

## Conclusion

Based on enrolment rates in our ITIP1 clinical trial, we identified specific areas, a core cluster, within Lilongwe with a high incidence of fast breathing pneumonia in children 2–59 months of age. Further examination of the environmental and socioeconomic features of this core cluster revealed that these areas are characterised by poverty, poor sanitation and possible crowding due to high population density. More research is needed to evaluate the impact of environmental and socioeconomic risk factors, along with vaccination status, on the development of childhood pneumonia.
